# DcR3, a new biomarker for sepsis, correlates with infection severity and procalcitonin

**DOI:** 10.18632/oncotarget.23736

**Published:** 2017-12-28

**Authors:** Liqin Gao, Bin Yang, Hairong Zhang, Qishui Ou, Yulan Lin, Mei Zhang, Zhenhuan Zhang, Sunghee Kim, Bing Wu, Zeng Wang, Lengxi Fu, Jingan Lin, Ruiqing Chen, Ruilong Lan, Junying Chen, Wei Chen, Long Chen, Hengshan Zhang, Deping Han, Jingrong Chen, Paul Okunieff, Jianhua Lin, Lurong Zhang

**Affiliations:** ^1^ Department of Laboratory Medicine, First Affiliated Hospital of Fujian Medical University, Fuzhou 350005, China; ^2^ Department of Radiation Oncology, University of Florida, Gainesville, Florida 32610, USA; ^3^ BioPowerTech, Tuscaloosa, Alabama 35406, USA; ^4^ Fujian Key Laboratory of Individualized Active Immunotherapy, Fuzhou 350005, China; ^5^ Key Laboratory of Radiation Biology of Fujian Province Universities, Fuzhou 350005, China

**Keywords:** plasma DcR3, sepsis, early diagnosis, correlation with procalcitonin, clinical value

## Abstract

Early diagnosis of sepsis is critical for successful treatment. The clinical value of DcR3 in early diagnosis of sepsis was determined in a dynamic follow-up study. Alterations in plasma levels of DcR3, PCT, CRP, and IL-6 were measured by ELISA and compared among patients with sepsis (*n* = 134), SIRS (*n* = 60) and normal adults (*n* = 50). Correlations and dynamic patterns among the biomarkers, APACHE II scores, clinical outcomes, and pathogens were also examined. Plasma DcR3 was significantly increased in sepsis compared to SIRS and normal adults (median 3.87 *vs.* 1.28 and 0.17 ng/ml). The elevated DcR3 could be detected in 97.60% sepsis patients 1–2 days prior to the result of blood culture reported. For diagnosis of sepsis, the sensitivity was 97.69% and specificity 98.04%; and for differential diagnosis of sepsis from SIRS, the sensitivity was 90.77% and specificity 98.40%. DcR3 level was positively correlated with severity of sepsis (*r*_s_ = 0.82). In 41 patients who died of sepsis, DcR3 elevated as early as 1–2 days before blood culture and peaked on day 3 after blood culture performed. In 90% of sepsis patients, the dynamic alteration pattern of DcR3 was identical to that of PCT, while pattern of 10% patients differed in which clinical data was consistent with DcR3. In 13% sepsis patients, while PCT remained normal, DcR3 levels were at a high level. DcR3 levels had no difference among various pathogens infected. DcR3, a new biomarker, will aid in early diagnosis of sepsis and monitoring its outcome, especially when sepsis patients were PCT negative.

## INTRODUCTION

Sepsis, the most common complication of the end-stage of many diseases, such as cancer (especially after chemotherapy exhausting the defense immune system), cardiovascular dysfunction, trauma, shock, infections, aging, etc. has a mortality rate up to ∼30% [1]. Because each hour that treatment is delayed increases mortality rates by 5–10%, early diagnosis and monitoring of treatment effectiveness are the keys to saving patients’ lives [2–3]. The most commonly used biomarkers for sepsis are procalcitonin (PCT), C reaction protein (CRP) and interleukin 6 (IL-6) [4–8]. CRP and IL-6 are unspecific pro-inflammatory molecules that increase in many acute diseases, including infection [9–13]. PCT, relatively specific to sepsis, is suspected to be an immunomodulator or chemoattractant [14–16].

The tumor necrosis factor receptor super-family (TNFR) is upregulated in response to stress to eliminate the damaged cells [17]. To maintain homeostasis, molecule with an opposite effect, such as DcR3, (a soluble decoy receptor 3 of the TNFR family with a binding domain without a signal transduction domain, 18), is upregulated to block the function of three pro-apoptotic molecules, such as Fas L [18], LIGHT [19] and TL1A [20], and serve as modulators or protective factors [21].

We speculated that circulating DcR3 might alter with the severity of sepsis, thereby serving as a good biomarker. Thus, we were the first to set up a quantitative enzyme-linked immunosorbent assay (ELISA) for DcR3 [22] and then to measure the plasma levels of DcR3 in normal adults and patients with systemic inflammation response syndrome (SIRS) or sepsis [23–24]. Our data suggested that plasma DcR3 is highly upregulated during sepsis and could be used to distinguish SIRS from sepsis [23–24]. This critical information could help guide treatment using either steroid hormones or antibiotics. However, in our previous studies, due to (1) the lack of side-by-side follow-up comparisons of DcR3 with commonly used biomarkers for sepsis, such as PCT, IL6 and CRP; and (2) the lack of a follow-up study of DcR3 with the course of sepsis, the value of DcR3 in clinical practice cannot be established.

In this follow-up cohort study, we advanced our previous work by following-up and comparing the plasma levels of DcR3, PCT, CRP, and IL-6 in 134 sepsis patients from the time when sepsis was suspected to 5–7 days after a pathogen-positive blood culture. The data were compared and analyzed for the alteration patterns and the correlation among these biomarkers, APACHE II scores, clinical outcomes and types of pathogens. The goal of this follow-up cohort study is to better understand and utilize DcR3 as a new biomarker for the early diagnosis of sepsis, monitoring treatment effectiveness and outcomes of sepsis.

## RESULTS

### General characteristics of patients with sepsis

Table 1 summarizes the clinical characteristics of a total of 50 normal adults, 60 SIRS (blood culture negative of pathogens) and 134 sepsis patients (as confirmed by pathogen-positive blood culture). The age and sex were relatively matched. The trauma, stroke, surgical operation on brain and chest were the main causes of SIRS, while some SIRS transition into sepsis, some of sepsis were due to chronic diseases of lung, liver, heart and kidney. All 134 sepsis patients were followed for DcR3, PCT, CRP, and IL-6 levels during the whole course of hospitalization.

**Table 1:** Clinical characteristics of subjects studied

	*N*	Age (mean ± SD)	Male (*n*)	Female (*n*)	Pathogen in blood culture	Sites of diseases		
**Normal adults**	50	47.7 ± 25.4	27 (54.0%)	23 (46.0%)	Negative			
**SIRS**	60	53.8 ± 18.6	37 (61.7%)	23 (38.3%)	Negative	Head trauma		4 (6.7%)
						Multiple trauma		11 (18.3%)
						Stroke		16 (26.6%)
						Brain tumor		4 (6.7%)
						Pancreatitis		3 (5.0%)
						Bleeding		2 (3.3%)
						Leukemia		4 (6.7%)
						SLE etc		8 (13.3%)
						Lung cancer		4 (6.7%)
						Heart failure		4 (6.7%)
**Sepsis**	134	56.7 ± 20.4	79 (59.0%)	55 (41.0%)	Positive	**Site of original infection** (32 with cancer)		
						Head/neck		20 (14.9%)
						Thorax		19 (14.2%)
						Abdomen		43 (36.6%)
						Pelvic cavity		8 (6.0%)
						Arms and legs		9 (6.7%)
						Blood		5 (3.7%)
						Other sites		24 (17.9%)
						**APACHE II Score**		
						<15	54 (43.3%)	
						15–24	44 (32.8%)	
						>25	36 (26.9%)	
						**Outcome of Sepsis**		
						Survived	93 (69.4%)	
						Death	41 (30.6 %)	

Notice: SIRS represented 60 patients with systemic inflammatory response syndrome (caused by trauma, stroke, brain tumor, bleeding, pancreatitis, SLE etc) with pathogen negative in blood culture and met 2 of the 4 clinical criteria: (1) a body temperature of > 38°C or < 36°C; (2) a heart rate > 90 beats per min; (3) a respiratory rate > 20 breaths per minute or an arterial CO_2_ pressure of < 32 mm Hg; and (4) white blood cell counts of >12,000 cells/ml or < 4000 cells/ml or >10% immature forms; Sepsis represented 134 different infectious patients with pathogen positive in blood culture and met 2 of the 4 above clinical criteria.

### DcR3 is a new biomarker for sepsis

**Figure 1:**
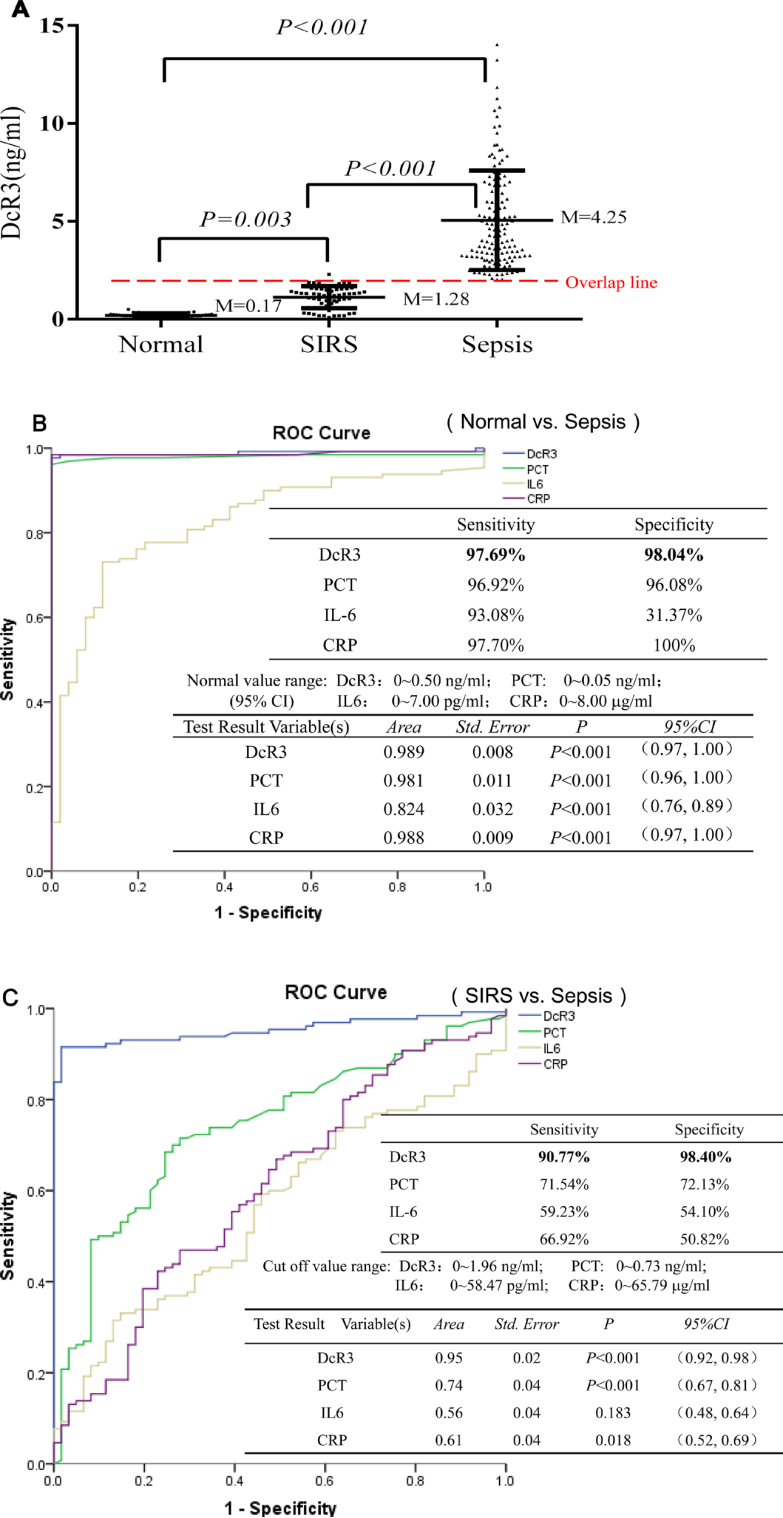
Levels of DcR3, PCT, IL-6, and CRP and ROC evaluation (**A**) the median value of plasma DcR3 in 50 normal adults, 60 SIRS patients, and 134 sepsis patients; (**B**) ROC evaluation of DcR3, PCT, IL-6, and CRP in normal *vs.* sepsis. The ROC evaluation was performed at cut-off values recommended by the scientific community of laboratory medicine with 95% CI (i.e., PCT 0∼0.05 ng/ml; IL6 0∼7.00 pg/ml; CRP: 0∼8.00 µg/ml), and DcR3 cutoff at 0∼0.50 ng/ml was based on mean + 2 standard deviation of 50 normal adults; (**C**) ROC evaluation of DcR3, PCT, IL-6, and CRP in SIRS *vs.* sepsis. ROC evaluation was performed at cut-offs of DcR3 0∼1.96 ng/ml; PCT: 0∼0.73 ng/ml; IL6: 0∼58.47 pg/ml; and CRP: 0∼65.79 µg/ml (mean + 2 standard deviation of SIRS measured), respectively.

The median value of plasma DcR3 was 0.17 ng/ml for normal adults, 1.28 ng/ml for SIRS patients, and 4.25 ng/ml for sepsis patients (Figure 1A). Receiver operating characteristic (ROC) analysis indicated that when the DcR3 cutoff was set at 0.50 ng/ml to distinguish the normal adults from those with sepsis, the sensitivity was 97.69% with a specificity of 98.04% (Figure 1B). SIRS patients were distinguished from sepsis patients at a DcR3 cutoff of 1.96 ng/ml (a value of 95% CI for SIRS tested) with a sensitivity of 90.77% and a specificity of 98.40%, better than the commonly used biomarkers of PCT, CRP, and IL-6 (Figure 1C).

Regarding the DcR3 value overlap among the groups, we found that: (1) there was not an overlap between the 50 normal adults and 134 sepsis patients; (2) 7 out of the 60 SIRS patients had high levels of DcR3 that were in sepsis range, while 2 out of the 134 sepsis patients had low levels of DcR3 in the SIRS range, thus the PPV (positive predictive value) is 94.96% and the NPV (negative predictive value) is 94.96%, which is good to distinguish SIRS from sepsis; (3) 3 out of the 50 normal adults had high levels of DcR3 in the SIRS range, while 10 out of the 60 SIRS patients had low levels of DcR3 in the normal range.

These data clearly suggest that DcR3 is a good index to distinguish among the normal, SIRS, and sepsis patients.

### DcR3 level was correlated with severity of sepsis

**Figure 2:**
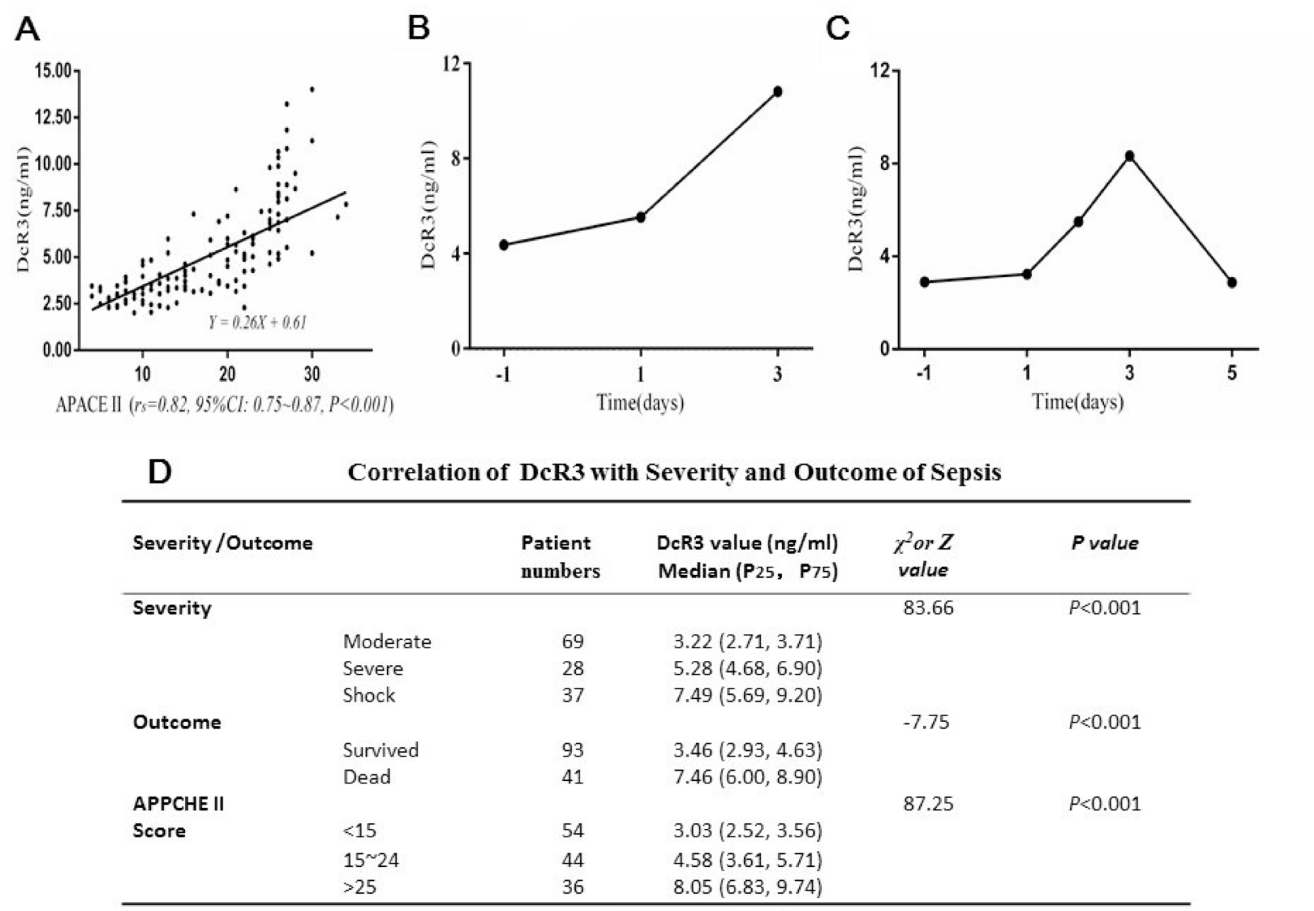
Correlation of DcR3 alteration with the severity and outcome of sepsis (**A**) correlation of DcR3 level with APACHE II scores (*r*_s_ = 0.82, 95% CI 0.75–0.87, *P* < 0.001). The respective equation Y = 0.26 X + 0.61 showed that the slope was 0.26 and the intercept 0.61; (**B**) in 13 out of 28 sepsis deaths, the typical alteration pattern of DcR3 continuously rose until death; (**C**) in 15 out of 28 sepsis deaths, the DcR3 alteration pattern reached a peak within 3 days at the time when sepsis was suspected or blood culture was carried out and then decreased before death; (**D**) the distributions of P_25_ (low 25% percentile) and P_75_ (high 75% percentile) in sepsis patients with different clinical outcomes and APACHE II scores. The outcome was represented by Z, while the severity and APACHE II score of sepsis was represented by χ^2^.

The correlation between DcR3 and the severity of sepsis presented by APACE II score was assessed with a nonparametric spearman correlation test. As Figure 2A shows, the *r*_s_ = 0.82 with 95% CI 0.75–0.87 (*P* < 0·001). The respective equation Y = 0.26x + 0·61 showed that the slope was 0.26 and the intercept 0.61. These data suggest a relatively strong positive correlation between DcR3 level and severity of sepsis, which was further confirmed by the fact that the DcR3 level increased from 3.22 ng/ml in the moderate symptom group to 5.28 ng/ml in the severe symptom group and to 7.49 ng/ml in the septic shock group (Figure 2D). In addition, the positive correlation was also reflected in the surviving and deceased cohorts (3.46 *vs.*7.46 ng/ml). When sepsis patients were divided based upon APACHE II scores, the group with scores < 15 had a median DcR3 level of 3.03 ng/ml, while scores around 15–24 were 4.58 ng/ml and scores > 25 were 8.05 ng/ml. These results indicated that the DcR3 level was well correlated with the severity of sepsis.

The individual DcR3 alteration patterns of during the course of sepsis were followed in 41 patients who eventually died. As shown in Figure 2B and 2C, when sepsis was suspected, even 1–2 days prior to a pathogen-positive blood culture, the DcR3 level was already elevated to > 3.5 ng/ml and peaked on day 3. There were two dynamic DcR3 alteration patterns: (1) in 13 patients (31.7%), DcR3 levels continuously rose, reaching their peak when the patients died (mean ± SD, 8.19 ± 2.96 ng/ml, Figure 2B); and (2) in 28 patients (68.3%), the DcR3 level peaked on day 3 after a blood culture was performed (mean ± SD, 7.47 ± 2.47 ng/ml), and then dropped to a lower level (Figure 2C). Twenty-five of these patients (89.3%) had a DcR3 level >5 ng/ml, whereas only 3 (10.7%) had a DcR3 level <5 ng/ml. These results indicate that once DcR3 reaches >5 ng/ml, the clinical outcome is poor.

All data suggest that DcR3 is likely to be an effective biomarker for: (1) early diagnosis of sepsis (before a pathogen-positive blood culture); (2) monitoring the effectiveness of treatment; and (3) predicting the clinical outcome of patients with sepsis.

### DcR3 reflected clinical alterations of sepsis as well as or better than PCT

**Figure 3:**
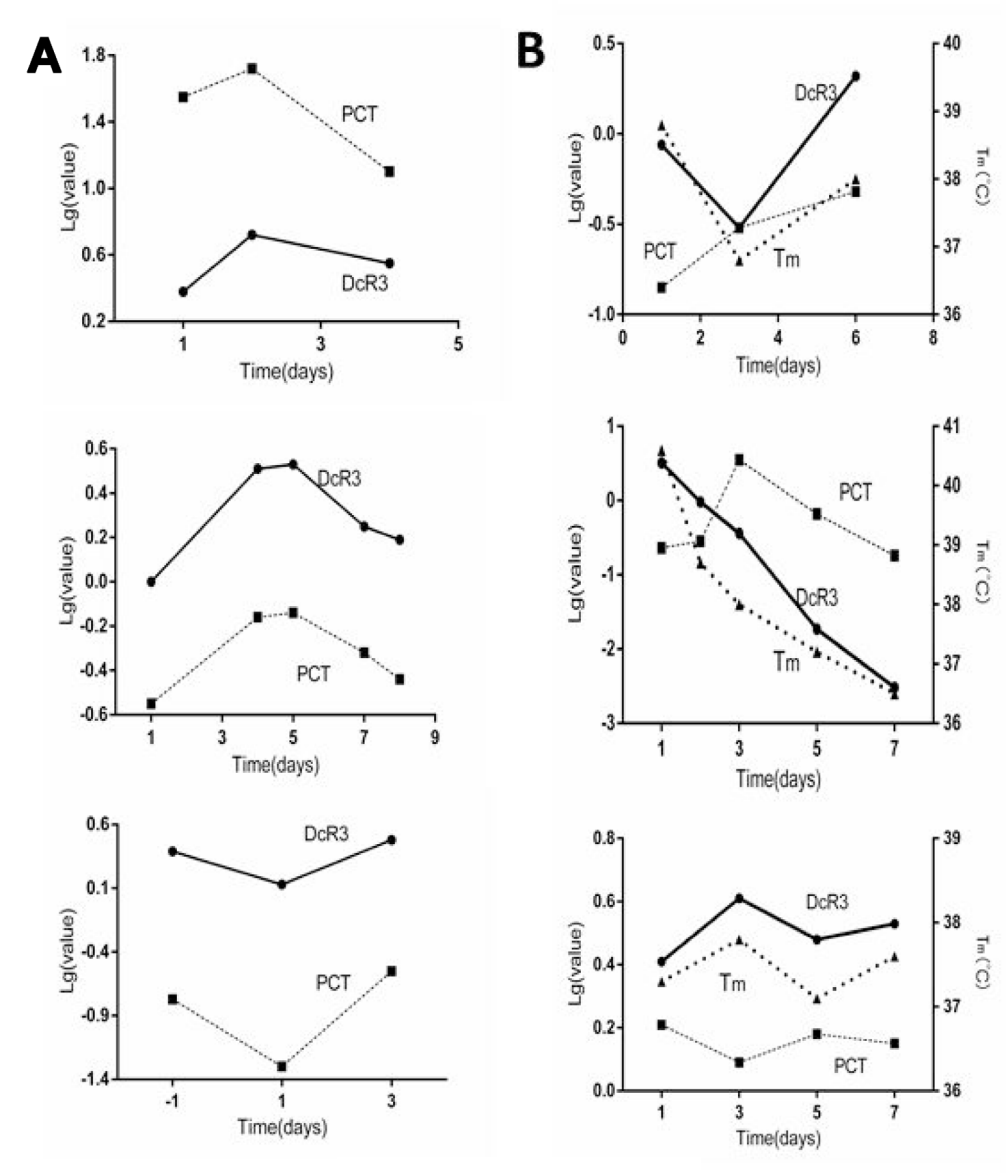
Comparison of DcR3 and PCT alteration patterns (**A** 1–3) in ∼ 90% sepsis patients, the alteration pattern of DcR3 was almost identical to that of PCT; (**B** 1–3) in ∼10% of sepsis patients, the alteration pattern tendency of DcR3 differed from that of PCT. The temperature alteration tendency of patients was similar to that of DcR3.

To determine if plasma DcR3 reflected the clinical alteration as effectively as PCT, most of the 134 patients were followed for 7–10 days during the course of disease. While the values of DcR3 and PCT were quite different, in order to show their alteration patterns with time in the same Figure, we utilized a semi-logarithmic chart. Figure 3A represents 3 typical patterns in which DcR3 and PCT had the same tendency of alteration with time, accounting for approximately 90% of the 134 patients. On the other hand, as Figure 3B shows, roughly 10% of patients had different alteration patterns between DcR3 and PCT; the body temperature (Tm) of that given patient was added to the Figure as a third parameter to judge whether DcR3 or PCT more accurately represented the clinical situation. The data showed that while DcR3 diverged from PCT, the Tm had the same alteration direction as DcR3, indicating that in some cases DcR3 reflects the clinical situation better than PCT. This was further confirmed by the fact that in some patients with sepsis, DcR3 was elevated while PCT remained normal (Table 2).

**Table 2:** 17 Sepsis patients had an elevated DcR3 while PCT was normal

Case number^#^	Testing time	DcR3^*^ ng/ml	PCT^**^ ng/ml
1	Blood culture day	2.04	0.07
2	Blood culture day	3.47	0.03
3	Blood culture day	1.29	0.07
4	One day before culture	7.64	0.03
5	One day before culture	1.17	0.02
5	Blood culture day	2.83	0.05
6	Blood culture day	2.37	0.02
6	8 days after culture	8.62	0.03
7	Blood culture day	1.20	0.02
8	Blood culture day	2.08	0.07
9	Blood culture day	1.78	0.06
10	Blood culture day	1.34	0.05
11	4 days after culture	2.33	0.07
12	One day before culture	1.17	0.03
13	Blood culture day	2.40	0.04
14	9 days after culture	1.02	0.06
15	2 days after culture	2.74	0.07
15	4 days after culture	3.04	0.06
16	2 days before culture	1.73	0.02
17	One day before culture	2.43	0.05

Notice: ^#^: the same case number represented the same patient at different test time-points. ^*^: DcR3 > 0.5 ng/ml was considered as abnormal; ^**^PCT > 0.07 ng/ml (board line) was considered as abnormal, <0.05 ng/ml was normal. In 17 out of 134 sepsis patients (12.69%), the DcR3 was at a high level (1.17–8.62 ng/ml) while PCT was about normal (0.02–0.07 ng/ml). Notably, the elevation of DcR3 occurred on 1–2 days before or on the day of blood culture ordered when sepsis was suspected while the results were not turned out yet.

### DcR3 elevated at early sepsis while PCT was normal

In most sepsis patients, DcR3 had the same tendency as PCT; however, as shown in Table 2, in 20 tests from 17 patients (∼12.68% of total patients), PCT levels were normal (median 0.05 ng/ml), whereas DcR3 levels were high (median 2.08 ng/ml). Notably, this occurred 1–2 days prior to or on the day of a pathogen-positive blood culture in 15 tests, while 8 out of 23 tests with PCT < 0.07 ng/ml were found 2.13 (6.3 ± 3.5) days after a pathogen-positive blood culture. The data suggested that in at least 10% of cases, DcR3 could detect sepsis while PCT was still at a normal level.

One sepsis patient had 4 tests on the day of blood culture and 2, 4, and 6 days after blood culture. His PCT levels were 0.12, 0.07, 0.06, and 0.03 ng/ml (normal in 3 out of 4 tests), whereas his DcR3 levels were 3.08, 2.73, 3.04, and 2.79 ng/ml (all at a high level). These results indicate that DcR3 might be a good alternative biomarker for sepsis patients whose PCT levels do not correlate well with sepsis. The combination of DcR3 and PCT might increase the detection rate of sepsis.

Taken together of above data, we did a NRI (Net Reclassification Improvement) test. Using pathogen-positive in blood culture as a gold standard to compare the diagnostic sensitivity and specificity of DcR3 with that of PCT, the result of NRI was 45.5 (*P* < 0.05), indication that the DcR3 improves the discrimination over PCT for sepsis.

### DcR3 could not reflect differences among infected pathogens

To determine if DcR3 level reflected any difference when patients were infected with different types of bacteria or fungi, sepsis patients were divided into gram-negative, gram-positive, and fungi groups. Table 3 shows that the median level of DcR3 was 4.33 ng/ml (P25 at 3.25 ng/ml, P75 at 6.76 ng/ml) in the gram-negative group (68.7%), 3.93 ng/ml (P25 at 2.92 ng/ml, P75 at 5.60 ng/ml) in the gram-positive group (23.9%), and 4.47 ng/ml (P25 at 2.39 ng/ml, P75 at 6.04 ng/ml) in the fungi group (6.7%). Data indicated that there was no correlation between DcR3 level and pathogen type (i.e., DcR3 could not distinguish the bacteria or fungi that caused sepsis).

**Table 3:** DcR3 level is not correlated with types of pathogens infected

	Case # (%)	Median of DcR3 (P25, P75)
**Gram-negative infections**	**99 (73.9%)**	**3.26 (2.30, 4.91)**
Escherichia coli	43 (32.1%)	3.09 (2.11, 4.66)
Enterococcus faecium	6 (4.5%)	5.54 (3.70, 8.64)
Klebsiella pneumoniae	19 (14.2%)	4.05 (3.07, 5.95)
Baumanii	8 (6.0%)	3.23 (2.52, 4.89)
Pseudomonas aeruginosa	3 (2.2%)	3.41 (2.29, 5.49)
Stenotrophomonas maltophilia	3 (2.2%)	3.86 (3.06, 6.15)
Others	17 (12.7%)	2.88 (2.86, 3.90)
**Gram-positive infections**	**26 (19.4%)**	**3.03 (2.36, 4.06)**
Staphylococcus aureus	15 (11.9%)	2.96 (2.48, 4.29)
Others	11 (7.5%)	3.04 (2.06, 3.71)
**Fungi infections**	**9 (6.7%)**	**3.34 (1.93, 4.18)**

## DISCUSSION

The pathogen-positive blood culture remains as a gold standard for the diagnosis of sepsis. While molecular biology has improved the turn-around time for pathogen detection in sepsis [25], the biomarkers that represent host responsiveness to the infected pathogens have been commonly used to speed up the early diagnosis of sepsis [1, 26]. We first developed an ELISA to measure the level of DcR3 [22], and then found that it is a good biomarker for sepsis [23, 24]. Our previous two studies were carried out either in Chinese [23], or in Caucasian [24], and the increased pattern of DcR3 in sepsis occurred in both races, indicating that DcR3 is likely to be a universal biomarker for sepsis for human beings.

However, in our previous studies we could not determine the clinical value of DcR3, since we did not carry out the following needed studies: (1) a side-by-side comparison of DcR3 with currently existing biomarker of sepsis, such as PCT, to determine their correlation and the advantages; (2) a detailed dynamic alteration pattern study of DcR3 in the whole course of sepsis by followed up for 7–11 days; (3) exploring the beneficial of testing both DcR3 and PCT; (4) the correlation between the DcR3 and pathogens.

First, using another large body of samples came from 134 Chinese sepsis patients who were diagnosed by blood culture positive of gram-negative or gram-positive bacteria or fungi, we further confirmed the clinical value of DcR3 for the early diagnosis of sepsis and for monitoring the severity and treatment outcome of sepsis.

Secondly, we found that sepsis patients had a much higher level of plasma DcR3 than normal adults and SIRS patients (Figure 1A), thereby allowing us to diagnose sepsis with a sensitivity and specificity equivalent to that achieved with PCT (Figure 1B and 1C). Notably, because DcR3 levels were moderate in SIRS and surged after sepsis (Figure 1A), we believe that DcR3 could be used to monitor the transition from SIRS to sepsis. Defining this transition is critical for treatment decision-making because SIRS requires the use of steroids, whereas sepsis relies on antibiotics. The mechanism underlying this phenomenon might be related to the protection properties of DcR3. This TNFR superfamily member is a decoy receptor that binds to three key proapoptotic molecules: Fas L [18], LIGHT [19], and TL1A [20]. Any stress that triggers the upregulation of proapoptotic molecules is likely to induce DcR3 to balance anti-apoptosis and anti-inflammation to ensure homeostasis [18, 20, 21, 27, 28].

In terms of alteration starting time, in most cases the plasma DcR3 level was elevated when sepsis is suspected and 1–2 days prior to a pathogen-positive blood culture. These results suggest that DcR3 could sensitively respond to the pathogenic infection at an early stage, thereby building the pathophysiological foundation for the use of DcR3 as an early diagnostic biomarker for sepsis.

In terms of alteration magnitude, the DcR3 level was well correlated with severity of sepsis, increasing as the disease worsened from moderate to severe to shock, and was positively correlated with the APACHE II score and mortality (Figure 2A and 2D). Studies have shown that lipopolysaccharides (LPS) from gram-negative bacteria or antigens of gram-positive bacteria could induce the production and release of DcR3 in a dose-dependent manner via toll-like receptor 4 (TLR-4) or TLR2 to activate extracellular signal-regulated kinase 1 and 2 (ERK1/2), c-Jun NH2-terminal protein kinase (JNK), and the transcription factor NF-kB [21, 29], which might account for the signal transduction path for the increase of DcR3 with the severity of sepsis.

In terms of alteration pattern with time, we found that two typical alteration patterns of DcR3 could predict treatment failure and mortality: (1) the level of DcR3 continued to increase (Figure 2B); and (2) the level reached higher than 5 ng/ml during the first 3 days when the blood culture was performed, although it later decreased (Figure 2C). The median value of DcR3 among the 93 survivors was 3.46 ng/ml with a P_25_ of 2.93 ng/ml and a P_75_ of 4.63 ng/ml compared to a median value of 7.46 ng/ml for the 41 patients who died with a P_25_ of 6.00 ng/ml and a P_75_ of 8.90 ng/ml (Figure 2D), suggesting that a high level of DcR3 achieved with time during the course of the disease might be a prognostic factor for poor clinical outcomes. It is likely that a high level of DcR3 represents a high stress factor in action that eventually leads to mortality. It has been reported that patients who succumb to acute respiratory distress syndrome (ARDS) have high DcR3 levels, regardless of their APACHE II scores, suggesting that DcR3 could be an independent predictor [27].

As Figure 3A shows, in approximately 90% of patients, the dynamic alteration pattern of DcR3 during the course of sepsis was very similar to that of PCT (the current best biomarker for sepsis); however, in ∼10% of cases, DcR3 and PCT exhibited different alteration patterns. In these patients, DcR3 reflected the clinical situation (as indicated by body temperature) better than PCT did (Figure 3B), indicating that DcR3 might reflect disease alterations.

Notably, DcR3 was upregulated in some sepsis patients who had a normal PCT level for unknown reasons. As shown in Table 2, in 20 tests for 17 sepsis patients (∼12.68%), PCT levels were normal (median 0.05 ng/ml), whereas the DcR3 levels were high (median 2.08 ng/ml). Among these tests, 14 (70%) were performed 1–2 days prior to or on the day a blood culture was performed when sepsis was first suspected. In some patients, the low pathogen levels could not stimulate PCT production, whereas DcR3 already responded, suggesting that DcR3 more sensitively reflected sepsis at a very early stage in some patients. The data suggest that a combination of DcR3 and PCT might more accurately detect early sepsis and monitor alterations in the course of sepsis than a single biomarker does.

The early identification of particular sepsis pathogens is also crucial to the treatment decision-making process. However, no difference in DcR3 levels between bacterial or fungal infections could be detected in blood cultures for 7 days, indicating that DcR3 could not reflect the pathogen cause of sepsis. The level of DcR3 might be related to the magnitude and course of sepsis, regardless of the pathogen. We did find that the DcR3 levels were lower in different viral infections than in bacterial or fungal infections (data unpublished). Bacterial and fungal pathogens could induce the production and release of DcR3 via different TLRs^21,29^ to counteract the elevated proapoptotic factors (e.g., FasL, LIGHT, and TL1A) and maintain homeostasis.

It is likely that pathogen-triggered endogenous DcR3 might not be enough to overcome the overwhelming effect of sepsis-induced proapoptotic factors. Several studies have explored the ability of exogenous DcR3 and its analogues to combat injury in models of infection or inflammatory diseases [19, 20, 30, 31]. Wortinger *et al.* demonstrated that FasL-induced accumulation of proinflammatory cytokines and neutrophils in the lung could be significantly reduced by the injection of an exogenous DcR3 analogue [32]. Similarly, an exogenous DcR3 analogue could reduce T-cell activation and enhance pulmonary bacterial clearance in mice challenged with streptococcus pneumonia [33]. More studies might shed new light on the effect of DcR3 on infection or inflammation.

While the biological function of increased PCT in sepsis remains unknown, the biological function of DcR3 is clear. The release of DcR3 is a counteraction to infection that can neutralize the elevated apoptotic molecules induced by pathogens to ensure homeostasis. Higher endogenous plasma DcR3 levels may indicate a greater magnitude of host damage and poor clinical outcomes, whereas lower levels could suggest less severe infections with a greater chance for survival. A continued increase in DcR3 might suggest that a current treatment is ineffective and that the treatment protocol should be altered.

Overall, the results indicate that plasma DcR3 is a sepsis biomarker that is equivalent to or better than PCT. When used in combination, DcR3 and PCT are likely to increase the positive detection rate for sepsis at a very early stage. DcR3 might also differentiate between SIRS and sepsis and predict the transition from SIRS to sepsis, allowing more efficacious use of steroids or antibiotics. Moreover, DcR3 can be used to assess the severity of sepsis and predict the outcome of the sepsis, thus enabling better decisions regarding treatment magnitude. Finally, it can be used to monitor treatment effectiveness, allowing clinicians to make rapid changes if necessary. More clinical studies of DcR3 are necessary and could lead to improvements in the survival rates for sepsis patients.

## MATERIALS AND METHODS

### Sample collection

Plasma from 50 normal adults, 60 patients with SIRS, and 134 patients with sepsis (a mean age of 40.4 ± 8.5, 56.2 ± 19.1 and 56.7 ± 20.4, respectively) was collected from Dec 2012 to Nov 2013, when the patients were admitted in the first affiliated hospital of Fujian Medical University at China. All patients with SIRS or sepsis were hospitalized due to complications of major surgery; trauma; heart, lung, liver, pancreas, kidney, or gastrointestinal diseases, or systemic infection.

The diagnosis of SIRS and sepsis were based on the American College of Chest Physicians/Society of Critical Care Medicine (ACCP/SCCM) consensus classification based on clinical symptoms and blood culture [34]. Each patient met at least 2 of the 4 clinical criteria: (1) a body temperature of > 38°C or < 36°C; (2) a heart rate > 90 beats per min; (3) a respiratory rate > 20 breaths per minute or an arterial CO_2_ pressure of < 32 mm Hg; and (4) white blood cell counts of > 12,000 cells/L or < 4000 cells/L or > 10% immature forms. The 7 day laboratory blood culture was the key to distinguish SIRS from sepsis [35, 36]. While the 60 SIRS patients had a negative of pathogens after 7 day blood cultures, the 134 sepsis patients were blood positive in either gram-negative, gram-positive bacteria or fungi in 12–72 hours after culture.

The starting sample of each patient used in this follow-up cohort study was collected at the point when patient had a fever and was suspected to be SIRS or sepsis and continuously collected until discharge. Mostly, the first plasma samples were collected 1–2 days prior to or on the day of blood culture, thereafter, samples were continuously collected 4, 6, 7, 10, or more days after a positive culture. Thus, most of patients had 4 to 8 serial samples tested.

For the comparison with currently common-used biomarkers, the patients’ samples were tested side-by-side with PCT that was mostly used as standard biomarker for suspected sepsis, and with IL6 as well as CRP that commonly used as referenced infectious biomarkers.

The plasma was divided into 0.5 ml/tube and stored at –80^o^C until analysis.

The severity of sepsis was evaluated based on APACHE II scores [34], clinical symptoms (moderate, severe, or shock) and outcome (survival or death). The pathogen of each patient was recorded.

59% of patients were men and 41% women. Normal adult samples were obtained from volunteers via blood bank donations.

This follow-up cohort study was approved by the institutional review board committee of the First Affiliated Hospital at Fujian Medical University (#2013-29).

### Measurement and analysis of plasma DcR3, PCT, CRP and IL6

A quantitative ELISA was used to measure DcR3 with methods previously reported by Chen *et al.* [22]. PCT and IL-6 were measured with a Cobas E601 Analyzer (Roche, Mannheim, Germany). CRP was measured using a CardioPhase hsCRP (Siemens, Marburg, Germany) with a BN II System (Siemens). All samples collected for DcR3 were measured in the same batch to avoid the inter-assay variation. The DcR3 standards were run simultaneously in the same assay for the calculation of unknowns. The intra-CVs of assays were <5–10% and the inter-CVs <8–15%.

The normal range of plasma DcR3 was determined based on the test of 50 cases of normal adults (95% CI, < 0.50 ng/ml, cut off value to distinguish the normal from sepsis), and the normal ranges of other biomarkers were adapted from the assay kits that were internationally recognized: PCT (95 or 99% CI < 0.05 or 0.09 ng/ml); IL6 (95% CI, < 7.00 pg/ml); and CRP (95% CI, < 8.00 mg/ml). The cutoff value to distinguish SIRS from sepsis used the 95% CI of SIRS tested.

The analysis of DcR3 and other biomarkers was focused on (1) comparison of their sensitivity and specificity to distinguish normal from sepsis and to differentiate SIRS from sepsis; (2) correlation of plasma DcR3 with the severity of sepsis; (3) the alteration pattern of DcR3 during the course of sepsis as comparison with PCT; (4) the difference between DcR3 and PCT, especially when PCT did not alter.

### Statistical analysis

The statistical analysis was performed using Statistical Package of Social Science (SPSS) version 13.0 for Windows. For age of studied groups, the mean and standard deviation were used, since they were normally distributed. For other variables studied, since they were not normally distributed, the median (25 quartile, 75 quartile) was used to express the majority range of levels. Comparisons among more than 3 variables were performed with a nonparametric Kruskal–Wallis test. Comparisons between 2 variables were analyzed with the nonparametric Mann–Whitney test. A receiver-operating characteristic (ROC) curve was used to evaluate the predictive value of DcR3 to diagnose sepsis. A nonparametric Spearman test was used to assess the correlation coefficient between 2 variables and to calculate the r_s_ (Spearman correlation index) with a 95% confidence interval (95% CI). 2-tailed *P* values less than 0.05 were considered to represent a statistically significant difference.

The positive predictive value (PPV) and negative predictive value (NPV) were calculated as: PPV = true positive/total positive × 100%; NPV = true negative/total negative × 100%. Net Reclassification Improvement (NRI) was determined according to the Peucina’s method [37], i.e. taken pathogen positive in blood as gold standard for diagnosis of sepsis, NRI = (sensitivity + specificity)_DcR3_ - (sensitivity + specificity)_PCT_, NRI > 0 as positive improvement.

## Abbreviations

DcR3: decoy receptor 3
PCT: procalcitonin
CRP: c-reactive protein
IL-6: interleukin 6
ELISA: enzyme-linked immunosorbent assay
SIRS: Systemic inflammatory response syndrome
APACHE II: Acute Physiology and Chronic Health Evaluation II
